# What mediates the link between childhood maltreatment and depression? The role of emotion dysregulation, attachment, and attributional style

**DOI:** 10.3402/ejpt.v7.32652

**Published:** 2016-10-26

**Authors:** Anna Schierholz, Antje Krüger, Jens Barenbrügge, Thomas Ehring

**Affiliations:** 1 Institute of Psychology, University of Münster, Münster, Germany; 2 Karl-Jaspers-Klinik, Wehnen, Germany; 3 Department of Psychology, LMU Munich, Munich, Germany

**Keywords:** Depression, childhood trauma, mediation, emotion dysregulation, attribution, attachment

## Abstract

**Background:**

Childhood maltreatment (CM) has been shown to be related to a severe and/or chronic course of depression. This study investigated which psychological processes mediate this relationship.

**Method:**

A large sample of acute or recovered depressed individuals (*N*=340) participated in an online survey assessing characteristics of depression, trauma exposure, and potential mediators (emotion regulation difficulties, attributional style, and attachment).

**Results:**

The experience of CM was related to more severe depression and more depressive episodes. In multiple mediation models, emotion dysregulation, a depressogenic attributional style, and avoidance in close relationships conjointly mediated the relationship between CM and depression severity as well as number of depressive episodes. However, a significant direct path between CM and depression characteristics remained. Exploratory analyses suggested that posttraumatic stress disorder symptom severity was an important additional mediator in our sample.

**Conclusions:**

Our findings provide preliminary evidence for psychological mediators between CM and depression that may be promising targets for interventions tailored for the treatment of depression in this subgroup.

**Highlights of the article:**

- Childhood maltreatment (CM) is related to more severe depression. The study investigates how this relationship can be explained.
- CM was related to difficulties in emotion regulation, relationships and thinking style, and symptoms of posttraumatic stress disorder.
- Statistical analyses showed that these four variables were also able to (partly) explain how CM is related to more severe depression.
- Treatment for depression in individuals who have experienced CM may need to directly target these variables.

Childhood maltreatment (CM) is highly prevalent and typically comprises repeated exposure to multiple forms of physical, sexual, and emotional abuse and/or neglect (Gilbert et al., 2009). It is associated with an increased risk for the development of different psychological problems, including major depressive disorder (MDD; Nelson, Klumparendt, Doebler, & Ehring, in press).

## CM and risk for depression

Evidence for a particularly strong association between CM and MDD comes not only from retrospective studies (e.g., Hovens et al., 2010; Wingo et al., 2010), but also from prospective longitudinal studies, even when controlling for influences of co-occurring adverse contextual factors (e.g., Brown, Cohen, Johnson, & Smailes, 1999; Widom, DuMont, & Czaja, 2007). Importantly, the experience of CM not only increases the risk for developing a depressive disorder at all, but also predicts the severity and course of the disorder. Specifically, recent meta-analytic findings show that CM is associated with a more severe and/or chronic course of depression and reduced response to treatment (Nelson et al., in press).

## Mediators of the link between CM and depression

In order to improve treatment for CM survivors suffering from depression, it appears important to better understand the processes mediating the link between maltreatment and depression. Heim, Newport, Mletzko, Miller, and Nemeroff (2008) suggest the existence of a biologically distinguishable subtype of depression as a function of CM. Specifically, depression following CM is supposed to be characterized by specific neuroendocrine and neuroanatomical changes (e.g., glucocorticoid resistance, immune activation, and reduced hippocampal volume) that reflect sensitization of central stress response systems. These changes are suggested to modulate the individual's competence to adapt to stressful life events, thereby increasing vulnerability to depression. This view is supported by empirical evidence showing that the relationship between CM and later depression is mediated by processes indicative of a sensitization of central stress response systems (e.g., Heim et al., 2008; Wiersma et al., 2011). Surprisingly few studies to date have looked at *psychological* processes that may mediate the association between early maltreatment and depression later in life. This study investigates three potential mediators, namely, emotion regulation difficulties, attachment, and attributional style. These variables were chosen based on the following criteria: (1) evidence for relationship with CM, (2) evidence for relationship with depression, (3) existing theoretical explanation for this variable as mediator, and (4) suitable for online assessment.

### Emotion regulation difficulties

One psychological process that may be related to the neurobiological alterations described above is emotion regulation. Indeed, there is emerging evidence supporting the role of emotion regulation difficulties as a mediator between CM and depression (Hopfinger, Berking, Bockting, & Ebert, 2016). In addition, indirect evidence comes from studies showing that CM leads to emotion regulation difficulties, including problems understanding and labeling affective states, low emotional acceptance, and preferential usage of maladaptive emotion regulation strategies (Cicchetti & Toth, 2005; Cook et al., 2005). Additionally, emotion regulation difficulties have been shown to be associated with depression (Joormann & Siemer, 2014).

### Attachment

Several authors suggest that CM contributes to depression vulnerability through disturbances in early attachment relationships (Goodman & Brand, 2009). Previous research has yielded strong and consistent support for an association between child maltreatment and insecure attachment in infants (Cicchetti & Toth, 2005; Cook et al., 2005; Stovall-McClough & Cloitre, 2006). Early attachment styles, in turn, are suggested to determine behavior, thoughts, and feelings in close interpersonal relationships in adulthood. In their influential model, Brennan, Clark, and Shaver (1998) conceptualize insecure attachment styles in adulthood as varying on two dimensions: anxiety and avoidance. Avoidance comprises the tendency to elude closeness and intimacy in relationships, whereas anxiety refers to feelings of insecurity and deficiency in close relationships. There is some evidence showing an association between CM and insecure attachment in adulthood (Styron & Janoff-Bulman, 1997).

Similarly, several studies have shown strong links between insecure attachment and risk for depression (Fonagy et al., 1996; Joiner & Timmons, 2002). However, despite strong indirect evidence, we are not aware of studies that have conducted formal mediation analyses testing the proposed associations between CM, attachment, and adult depression.

### Attributional style

According to Abramson, Metalsky, and Alloy (1989), experiences of hopelessness can lead to depressogenic inferential styles that are characterized by a tendency to attribute negative life events to internal, stable, and global causes. There is an ample evidence that this particular attributional style is an enduring cognitive risk factor that interacts with acute stressors and reliably predicts depressive reactions following stressful life events (Alloy, Abramson, Safford, & Gibb, 2006; Joormann, 2009). Furthermore, Wiersma et al. (2011) found evidence that hopelessness and an external locus of control are associated with a more chronic course of depression. Although depressogenic attributional style has been suggested as mediating the relationship between maltreatment and depression on a theoretical level (Goodman & Brand, 2009), no study has directly examined this relationship yet.

## This study

In this study, proposed mediators of the relationship between CM and depression were assessed simultaneously in a large group of individuals with a depressive disorder. Based on previous research and theoretical views described above, we hypothesized (1) that CM is associated with more severe depression and a higher number of depressive episodes, and (2) that CM as well as severity and course of depression are associated with the proposed mediators emotion regulation difficulties, maladaptive adult attachment, and depressogenic attributions. (3) We predicted that in a multiple mediation model, the set of proposed mediators reveal a substantial indirect effect in explaining the relationship between CM and depression severity on the one hand and number of depressive episodes on the other hand.

## Methods

### Participants

The study was conducted as a web-based survey. Participants were recruited via online communities and self-help organizations for depressed individuals. Furthermore, leaflets were posted at university buildings, clinical information centers, and general practitioners’ and psychotherapists’ offices in Münster (Germany). In total 1,920 individuals retrieved the first page of the survey. One thousand fifty-four individuals agreed to participate after reading the detailed study information on the first page and completed questions assessing eligibility criteria. Inclusion criteria were age between 18 and 65 years, fluency in German, and a suspected lifetime diagnosis of MDD according to the depression module of the Patient Health Questionnaire (PHQ-9; Kroenke, Spitzer, & Williams, 2001). Exclusion criteria were screening positive for a history of manic or psychotic symptoms, or current frequent thoughts about suicide or self-harm (*nearly every day* according to Item 9 of the PHQ-9).

Individuals were excluded if they did not meet eligibility criteria (*n=*346, 32.8%), did not complete the whole survey (*n=*307, 29.1%), or denied final consent at the end of the survey (*n=*7, 0.7%). In total, 394 participants (37.4%) were eligible, provided complete data, and gave final consent for data use on the last page of the survey. Participants were excluded from data analysis because they selected the option “not specified” for more than 40% of Childhood Trauma Questionnaire (CTQ) items (*n=*11), or because they had never been in a romantic relationship and, therefore, could not fill in the attachment questionnaire (*n=*43). The final sample comprised 340 participants (82.4% women; age: *M*=36.1 years, SD=12.0).

### Materials

#### Depression

The depression module of the *PHQ-9* (Kroenke et al., 2001; German version: Löwe, Spitzer, Zipfel, & Herzog, 2002) was used to screen for a diagnosis of current and past MDD and to assess symptom severities. The PHQ-9 has shown good psychometric properties (Kroenke et al., 2001; current study: α=0.87), including high sensitivity and specificity when validated against structured clinical interviews (Gilbody, Richards, Brealey, & Hewitt, 2007).The number of depressive episodes experienced was measured by an additional question following the PHQ-9.

#### Childhood maltreatment

CM was assessed with the short version of the CTQ (Bernstein et al., 2003; German version: Wingenfeld et al., 2010). It captures the severity of physical, sexual, and emotional abuse as well as physical and emotional neglect using 25 items. The short CTQ shows good psychometric properties (Klinitzke, Romppel, Häuser, Brähler, & Glaesmer, 2012; Wingenfeld et al., 2010). The total score was used in this study (α*=*0.93).

#### Proposed mediators

Emotion regulation difficulties were measured by the Difficulties in Emotion Regulation Scale (DERS; German version: Ehring, Fischer, Schnülle, Bösterling, & Tuschen-Caffier, 2008; Gratz & Roemer, 2004). The DERS measures the flexibility and adaptiveness of emotion regulation, including (lack of) emotional awareness and clarity, (non-)acceptance of emotions, difficulties engaging in goal-directed behavior when distressed, impulse control difficulties, and limited access to emotion regulation strategies. Psychometric properties of the measure are good (Gratz & Roemer, 2004). The total score of all 36 items was used in this study (α*=*0.95).

The Experiences in Close Relationships Questionnaire (ECR; Brennan et al., 1998; German version: Neumann, Rohmann, & Bierhoff, 2007) served to assess quality of attachment in adult relationships on the two dimensions anxiety and avoidance. Neumann et al. (2007) confirmed good psychometric properties of the ECR in diverse samples. The sum score of each subscale was used in this study (α=0.91–0.93).

Depressogenic attributional style was measured using the Depressive Attributions Questionnaire (DAQ; Kleim, Gonzalo, & Ehlers, 2011). It is based on the hopelessness theory of depression (Abramson et al., 1989) and captures the tendency to attribute negative events to internal, stable, and global causes. Earlier research has shown good psychometric properties (Kleim et al., 2011). The total score was used in this study (α*=*0.93).

The Posttraumatic Diagnostic Scale (PDS; Foa, Cashman, Jaycox, & Perry, 1997; German version: Griesel, Wessa, & Flor, 2006) was used to assess the severity of PTSD symptoms according to DSM-IV-TR. The 17-item PDS possesses excellent psychometric properties (Griesel et al., 2006; this study α=0.91). The measure was originally included to better characterize the sample. However, in the process of data analyses, it was decided to include the PDS as an additional mediator (see below for details).

### Procedure

The study was approved by the local Research Ethics Committee and conducted in accordance with the declaration of Helsinki. The Unipark platform (www.unipark.com) was used to generate and launch the web-based, secured, and encrypted survey. For ethical reasons, in all questionnaires inquiring about traumatic experience, it was possible to refuse an answer by selecting the option “not specified.” After completing all questionnaires, participants were asked whether they still agreed to data usage and whether they had answered all questions in an honest manner. As required by the ethics committee, only participants who provided informed consent at the beginning of the study and agreed to data usage at the end of the survey were included in the analyses.

### Data analysis

Prior to statistical analysis, missing values on the CTQ were imputed *via* response-function-imputation (Sijtsma & Van der Ark, 2003) for 54 participants (15.9% of the final sample). Furthermore, descriptive statistics and distribution properties of the variables of interest were examined. It became obvious that answers to the variable *number of lifetime episodes of depression* were distributed in an acceptable way until 5 and afterwards biased toward numbers that can be divided by 5 (e.g., 10, 15, and 20). This accumulation is a strong indication of non-equidistance of the frequency data. The variable was therefore transformed into seven categories (one/two/three/four/five/six to ten/eleven and more episodes) and treated as ordered categorical data in all analyses.

To test Hypothesis 1, correlations between measures of CM severity (CTQ total score) and depression characteristics were estimated using Mplus 7.11. Because of the ordered categorical dependent variable (DV) *number of life-time depressive episodes*, a robust weighted least squares estimator (WLSMV) was used as recommended by Muthén and Muthén (2012). Furthermore, associations between CM, depression characteristics, and proposed mediators were analyzed in the same manner (Hypothesis 2). Each mediator showing significant associations with depression and CM in the previous analyses was considered in the following multiple mediation models that tested Hypothesis 3 using path analyses in Mplus. According to Mplus recommendations (Muthén & Muthén, 2012), the multiple mediation model with depression severity as continuous DV was conducted using a maximum likelihood estimator, whereby a WLSMV estimator was used for the path analysis with the number of depressive episodes as an ordered categorical DV. Furthermore, bootstrap analyses with bias-corrected confidence estimates on a 95% confidence level (Preacher & Hayes, 2008) were used in order to test significance of total, direct, and indirect effects. Bootstrap estimates were based on 10,000 bootstrap samples. The total indirect effect represents the difference between the total (*c*) and direct effects (*c’*) of the CTQ on depression characteristics. Its significant difference from zero indicates that the complete set of proposed mediators accounts for a substantial amount of the total effect of CTQ scores on depression characteristics. The total indirect effect can be subdivided into specific indirect effects to analyze the amount of each potential mediator to the total effect (c). In the multiple mediation models, intercorrelations between all proposed mediators were allowed.

Potential influences of age and gender were tested by including them simultaneously as covariates in each mediation model. As the pattern of findings remained unchanged, only the mediation models without covariates are reported in detail.

## Results

About 31.5% of the final sample met criteria for a current depressive episode (107 acute depressed individuals); the remaining 68.5% had suffered from at least one past depressive episode (233 recovered depressed individuals). The median of life-time episodes was four.

About 64.4% of the sample indicated to have experienced at least one traumatic event according to DSM-IV-TR criteria in their lives. For 54% of this subgroup, the index trauma was the experience of sexual and/or physical violence before the age of 14 (interpersonal trauma after the age of 14: 25%; non-interpersonal trauma: 21%). Participants’ responses on the PDS showed that 64% of individuals who indicated to have experienced any traumatic life event also reported symptoms that suggest a current DSM-IV diagnosis of PTSD according to the algorithm suggested by Ehring, Kleim, Clark, Foa, and Ehlers (2007). This proportion represents 41% of the total sample.

### Correlational analyses

Correlations between the relevant variables of the hypothesized mediation model are shown in Table 1. As predicted by Hypothesis 1, CM severity (CTQ) was significantly correlated with depression severity as well as the number of depressive episodes.

**Table 1:** Means, standard deviations, and WLSMV estimated correlations between childhood maltreatment, depression, and proposed mediators

Variable	*M*	SD	1	2	3	4	5	6	7
1. CTQ	51.39	18.58	1.00	0.21***	0.31***	0.27***	0.38***	0.12*	0.27***
2. PHQ-9	11.56	5.83		1.00	0.20***	0.59***	0.23***	0.26***	0.52***
3. Number of episodes	(ordinal)				1.00	0.25***	0.24***	0.05	0.20***
4. DERS	104.55	26.68				1.00	0.40***	0.40***	0.74***
5. ECR avoidance	61.93	22.53					1.00	−0.02	0.35***
6. ECR anxiety	71.18	22.28						1.00	0.47***
7. DAQ	31.75	14.06							1.00

CTQ=Childhood Trauma Questionnaire; PHQ-9=Patient Health Questionnaire; DERS=Difficulties in Emotion Regulation Scale; ECR=Experience in Close Relationships Questionnaire; DAQ=Depressive Attributions Questionnaire.

* *p*<0.05;

** *p*<0.01;

*** *p*<0.001.

Secondly, in line with Hypothesis 2, CTQ scores were significantly associated with all proposed mediators. In addition, scores on the DERS, ECR avoidance subscale, and DAQ were significantly correlated with depression severity and number of depressive episodes. However, scores on the ECR anxiety subscale were not significantly associated with the number of depressive episodes.

### Multiple mediation models

**Fig. 1:**
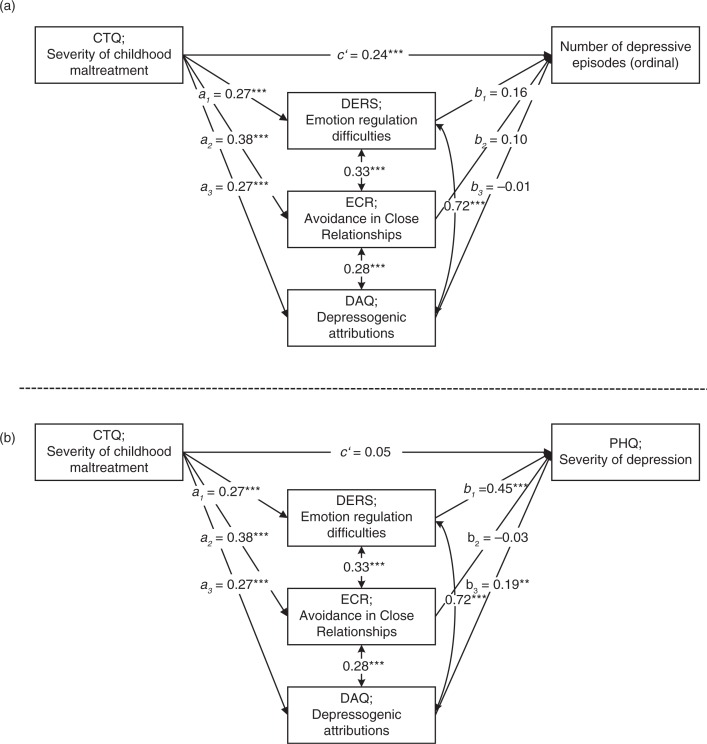
Standardized results for the direct paths of the multiple mediation models for number of depressive episodes (a) and Depression Severity (b) *Excluding* PDS Scores. *p<0.05; **p<0.01; ***p<0.001.

In the first path analysis, CTQ scores were the independent variable (IV) whereas the number of depressive episodes served as DV (Fig. 1a). Because the correlational analyses had shown scores on the DERS, ECR avoidance subscale, and DAQ to be most consistently associated with measures of CM and depression, they were included as potential mediators in the multiple mediation model.

The total effect of CM on the number of depressive episodes (*c path*) was significant (Table 2), which confirmed results of the correlational analyses described above. As indicated by the bootstrap confidence intervals, the total indirect effect of severity of CM on the number of depressive episodes through scores on the DERS, ECR avoidance subscale, and DAQ was also significant (Table 2), supporting Hypothesis 3. However, when examining the specific indirect effects of each potential mediator, no significant effect of a single mediator was found (Table 2). In addition, the direct effect of the CTQ on the number of depressive episodes was still significant (Fig. 1a), suggesting that the set of proposed mediators only partially mediated the relationship between childhood trauma and the course of depression.

**Table 2:** Standardized bootstrap results for total, total indirect, specific indirect, and direct effects of the multiple mediation models excluding PTSD symptom severity

				BC 95% CI	
				
	Std. point estimate	SE	*p*	Lower	Upper
DV=number of episodes					
Total (*c*)	0.31	0.05	<0.001	0.21	0.42
Total indirect	0.08	0.02	0.002	0.03	0.12
Specific indirect					
DERS	0.04	0.02	0.06	−0.01	0.09
ECR avoidance	0.04	0.02	0.12	−0.01	0.08
DAQ	−0.01	0.02	0.86	−0.05	0.04
Direct (*c’*)	0.24	0.06	<0.001	0.12	0.35
DV=depression severity					
Total (*c*)	0.21	0.05	<0.001	0.11	0.31
Total indirect	0.16	0.04	<0.001	0.09	0.23
Specific indirect					
DERS	0.12	0.03	<0.001	0.06	0.18
ECR avoidance	−0.01	0.02	0.48	−0.05	0.02
DAQ	0.05	0.02	0.02	0.01	0.10
Direct (*c’*)	0.05	0.05	0.29	−0.04	0.14

DV=Dependent Variable; BC=Bias Corrected; CI=Confidence Interval; Std.=Standardized; based on 10,000 bootstrap samples; multiple mediation models of X → M → Y (where X is scores on the Childhood Trauma Questionnaire, CTQ; M is scores on the three proposed mediator variables: Difficulties in Emotion Regulation Scale, DERS; avoidance subscale of the Experiences in Close Relationships Questionnaire, ECR; Depressive Attributions Questionnaire, DAQ; and Y is number of lifetime depressive episodes, or the severity of depressive symptoms, respectively).

In a second step, a multiple mediation model containing the same independent and mediating variables but current depression severity as DV was conducted (Fig. 1b). The total effect of severity of CM on severity of current depression (*c path*) was significant (Table 2). Furthermore, the bootstrap confidence intervals indicated that the total indirect effect of severity of CM on depression severity was significant (Table 2). The DERS and DAQ emerged as specific variables significantly mediating the relationship between severity of CM and current depression severity, whereas the ECR avoidance subscale did not contribute additionally to the indirect effect. Importantly, the direct effect (*c’*) of CTQ on depression severity was no longer significant after considering the indirect effects of the proposed mediators (Table 2).

The pattern of findings for both mediation models did not change when entering the two potential covariates, gender and age, into the analyses.

### Exploratory analyses: PTSD symptom severity as an additional mediator

As the direct effect of the CTQ on the number of depressive episodes (*c’*) was still significant after considering the indirect effects of the proposed mediators, further unplanned and exploratory analyses were conducted exploring PTSD symptom severity as an additional mediator. PDS scores were significantly correlated with all relevant variables included in the multiple mediation model (Table 3). Because scores on the PDS were only available for participants who had experienced at least one traumatic event, the analyses could only be conducted for this subgroup (*n=*219; 83.1% women; age: *M=*37.2 years, SD*=*12.2; see Table 3 for intercorrelations between variables of interest for this subgroup only).

**Table 3:** Means, standard deviations, and WLSMV estimated correlations between severity of childhood maltreatment, characteristics of depression, proposed mediators, and PTSD symptom severity

Variable	*M*	SD	1	2	3	4	5	6	7
1. CTQ	56.76	19.43	1.00	0.24***	0.25***	0.27***	0.34***	0.24***	0.46***
2. PHQ-9	11.89	5.76		1.00	0.28***	0.61***	0.19**	0.55***	0.53***
3. Number of episodes	(ordinal)				1.00	0.29***	0.18*	0.18**	0.33***
4. DERS	106.90	26.93				1.00	0.40***	0.75***	0.52***
5. ECR avoidance	64.64	22.47					1.00	0.32***	0.37***
6. DAQ	33.01	13.83						1.00	0.45***
7. PDS	22.87	12.06							1.00

CTQ=Childhood Trauma Questionnaire; PHQ-9=Patient Health Questionnaire; DERS=Difficulties in Emotion Regulation Scale; ECR=Experiences in Close Relationships Questionnaire; DAQ=Depressive Attributions Questionnaire; PDS=Posttraumatic Stress Diagnostic Scale.

* *p*<0.05;

** *p*<0.01;

*** *p*<0.001.

**Fig. 2:**
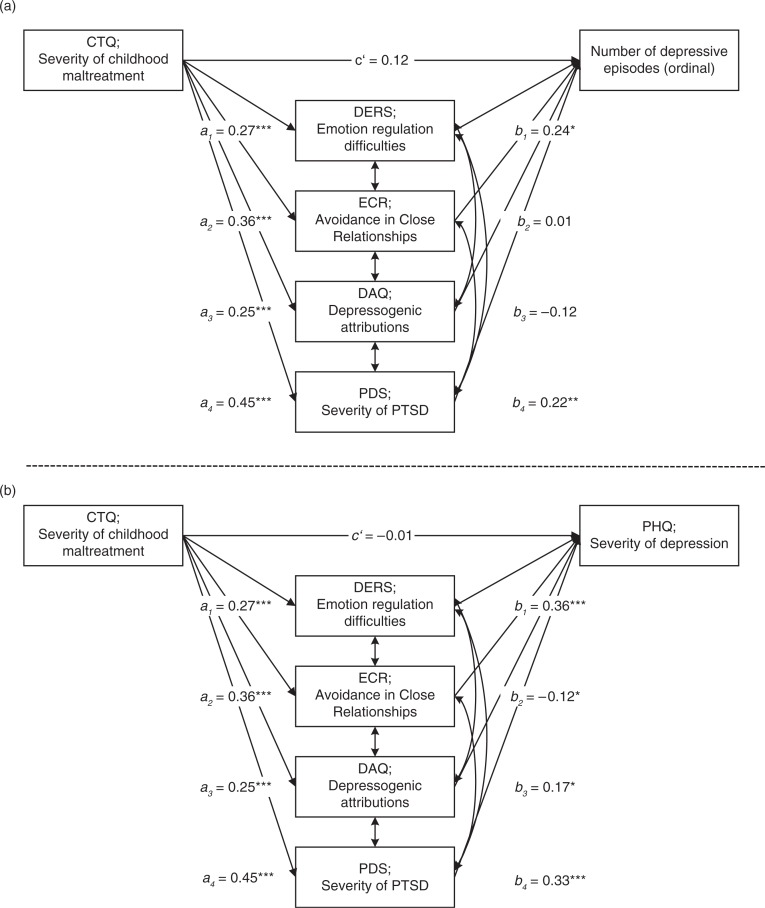
Standardized results for the direct paths of the multiple mediation models for number of depressive episodes (a) and Depression Severity (b) *Including* PDS Scores. *p<0.05; **p<0.01; ***p<0.001.

The first extended multiple mediation model with the number of depressive episodes as DV showed a significant total indirect effect for the four mediators (Table 4). This total indirect effect was mainly driven by the only two significant specific indirect effects through emotion regulation difficulties and PTSD symptom severity. Importantly, the direct effect from CTQ to the number of depressive episodes was no longer significant (for detailed results, see Table 4 and Fig. 2a).

**Table 4:** Standardized bootstrap results for total, total indirect, specific indirect, and direct effects of the multiple mediation models including PTSD symptom severity

				BC 95% CI	
				
	Std. point estimate	SE	*p*	Lower	Upper
DV=number of episodes					
Total (*c*)	0.25	0.07	<0.001	0.12	0.39
Total indirect	0.14	0.04	0.001	0.06	0.21
Specific indirect					
DERS	0.07	0.03	0.03	0.01	0.13
ECR avoidance	0.01	0.03	0.93	−0.05	0.05
DAQ	−0.03	0.03	0.23	−0.09	0.02
PDS	0.10	0.04	0.02	0.02	0.18
Direct (*c’*)	0.12	0.08	0.14	−0.04	0.28
DV=depression severity					
Total (*c*)	0.24	0.06	<0.001	0.11	0.37
Total indirect	0.25	0.05	<0.001	0.14	0.35
Specific indirect					
DERS	0.10	0.03	0.003	0.03	0.16
ECR avoidance	−0.04	0.02	0.04	−0.09	−0.01
DAQ	0.04	0.02	0.07	−0.01	0.09
PDS	0.15	0.04	<0.001	0.08	0.22
Direct (*c’*)	−0.01	0.06	0.93	−0.11	0.10

BC=Bias Corrected; CI=Confidence Interval; Std.=Standardized; based on 10,000 bootstrap samples; multiple mediation model of X → M → Y (where X is scores on the Childhood Trauma Questionnaire, CTQ; M is scores on the three proposed mediator variables: Difficulties in Emotion Regulation Scale, DERS; avoidance subscale of the Experiences in Close Relationships Questionnaire, ECR; Depressive Attributions Questionnaire, DAQ; Posttraumatic Diagnostic Scale, PDS; and Y is current depression severity as measured by Patient Health Questionnaire, PHQ-9).

Similarly, in the second extended multiple mediation model using depression severity as the DV there was also a significant total indirect effect through the four mediators found (Table 4). The direct effect of CTQ on depression severity was not significant anymore in this model. The indirect effect emerged as being primarily driven by PTSD symptom severity, with DERS and DAQ also showing significant but somewhat lower effect (for detailed results, see Table 4 and Fig. 2b).

## Discussion

Past research has consistently shown that CM increases the risk of developing depression, but is also associated with higher depressive symptom severity and a more severe course of the disorder (Nelson et al., in press). This study replicates and extends these findings in a sample of acute and recovered depressed individuals. As predicted, the CM was associated with the severity of depression and the number of lifetime depressive episodes.

From a theoretical point of view, it appears interesting to investigate the processes mediating the link between CM and depression. In addition, this is also highly relevant from a clinical perspective as it may provide information on promising targets for innovative interventions offered specifically for CM survivors suffering from depression. This study investigated the role of emotion regulation difficulties, attachment, and attributional style as three possible psychological mediators.

### Relationship between CM and potential mediators

CM was significantly and substantially associated with all proposed mediators. Depressed individuals who had been exposed to high levels of CM showed more severe impairments in understanding and accepting their emotions, engaging in goal-directed behavior, controlling impulsive behavior when experiencing negative emotions, and using appropriate emotion regulation skills. This finding is consistent with earlier findings showing a close association between CM and emotion regulation difficulties (Cook et al., 2005; Coyle, Karatzias, Summers, & Power, 2014; Ehring & Quack, 2010). In addition, participants who had experienced more severe CM reported a larger tendency to elude closeness and intimacy in relationships (avoidance) and to attribute negative life events to internal, stable, and global causes (depressogenic attributional style). Both results support previous research and theories suggesting an association between CM and insecure attachment as well as dysfunctional attributional style (e.g., Sarin & Nolen-Hoeksema, 2010; Styron & Janoff-Bulman, 1997).

### Mediation analyses

Our key hypothesis was that emotion regulation difficulties, avoidant attachment, and a depressogenic attributional style mediate the association of CM and the severity and course of depression. This hypothesis was tested in two multiple mediation models with the DVs depressive symptom severity and number of depressive episodes, respectively. Results supported the hypothesis in that the complete set of mediators had significant indirect effects in explaining the relationship between severity of CM and depression severity as well as the number of episodes. The directions of the *a* and *b* paths were consistent with the interpretation that more severe CM leads to more difficulties in emotion regulation, more avoidance in close relationships, and more depressogenic attributions, which in turn leads to more severe depression.

It should be noted that results were more clear-cut for depression severity than for the number of episodes experienced. When looking at depression severity, the three psychological processes fully mediated the relationship between CM and severity of depression. However, for the number of episodes as DV, the direct path from CM to depression (*c’*) remained significant. In addition, emotion regulation difficulties and depressogenic attributional style were identified as significant specific mediators for depression severity, whereas no significant specific indirect effect was found for any of the mediators for the number of episodes. This suggests that additional mediators not included in these models may play a role. Note that evidence from other lines of research also suggests that different processes are involved in the development of episodes experienced early versus later in the illness course, including a possible role of kindling processes (Monroe & Harkness, 2005).

### The role of PTSD symptom severity

Unexpectedly, a large proportion of participants in our sample showed at least some degree of PTSD symptoms. It appeared conceivable that higher depression severity and a more severe course of depression in participants with CM may at least partly be related to PTSD symptoms experienced by this group. In order to test this idea, the two mediation analyses were repeated, entering PTSD symptom severity as an additional mediator. Significant indirect effects were found in both analyses, and the direct path from CM to depression was rendered non-significant. The significant indirect effects were found to be primarily driven through PTSD symptom severity. Emotion regulation difficulties could be identified as the only potent mediator over and above PTSD.

The high prevalence of PTSD symptoms in our sample was unexpected, as participants were merely recruited based on their depression status, and neither PTSD nor CM were mentioned during recruitment. A selection bias in the recruitment phase therefore appears unlikely. As many earlier studies in this area did not systematically assess PTSD, it remains unclear whether the high prevalence of PTSD symptoms in our sample is unusual.

### Possible implications

Our findings raise a number of interesting hypotheses that should directly be tested in future research. First, the high intercorrelation between the mediators raises the question whether these are different or rather similar constructs. From a statistical point of view, collinearity of mediators is a common phenomenon in multiple mediation models because mediators are assumed to have a common cause (Preacher & Hayes, 2008). However, this renders the interpretation of indirect effects for specific mediators less informative than the total indirect effect. In addition, theory predicts the different mediators to represent independent psychological constructs that are nevertheless related in specific ways. For example, as emotion regulation is typically learned in interaction with key caregivers, CM can be expected to impair the development of adaptive emotion regulation at least partly *via* its negative impact on attachment processes (Ehring & Quack, 2010). In order to better understand the effect of CM on emotional, cognitive, interpersonal, and biological vulnerabilities, more specific theoretical models regarding the interplay of these different processes need to be developed and tested using longitudinal designs.

Second, a number of explanations for the finding of PTSD as a strong mediator between CM and depression are conceivable. From a traditional disorder-focused view, this finding is not entirely surprising as there is a substantial symptom overlap between MDD and PTSD, which may inflate their relationship in our data. In addition, PTSD may be a primary response to CM that amplifies the severity and maintenance of secondary depressive symptoms later in life.

From a transdiagnostic perspective, on the other hand, the data may suggest that there are two different pathways linking CM to depression later in life. *Via* the first pathway, CM impairs the development of self-regulation on an emotional, interpersonal, cognitive, and psychobiological level, which in turn increases vulnerability for depression. The second pathway, on the other hand, means that the trauma of CM itself has not been processed in an adaptive way, leading to intrusive memories, negative trauma-related cognitions, and high emotional and/or physiological reactivity to trauma reminders, which in turn triggers and maintains the symptoms of depression. The crucial distinction between the two pathways is the hypothesized role of CM for the processes maintaining current symptomatology. In Pathway 1, CM is merely a distal etiological factor, whereas in Pathway 2, CM is suggested to be re-experienced in the form of CM-related memories, thoughts, feelings, and/or behaviors. Based on the current data, this transdiagnostic view on the link between CM and depression remains highly speculative. However, if supported in future research it may have important clinical implications. For CM survivors suffering from depression who additionally show high levels of CM-related memories, thoughts, feelings, or behaviors, trauma-focused treatments aimed at processing the traumatic experience may be promising, even if they do not suffer from full-blown PTSD. For depressed CM survivors without these phenomena, on the other hand, interventions targeting emotion regulation, attachment, and/or attributional style appear more promising.

### Strengths and limitations

Our study shows a number of strengths, including the relatively large sample size. In addition, this is one of the first studies investigating multiple psychological mediators between CM and clinical characteristics of depression. On the other hand, some limitations are noteworthy. Firstly, because of the cross-sectional design, it could not be tested whether the mediators indeed preceded the outcome. Although, we think that a cross-sectional design is defendable as a first step, future research using longitudinal designs is necessary. In addition, future studies will need to assess potentially confounding variables in more detail. For example, avoidant attachment in adulthood may not only be the consequence of CM but other processes such as late-life trauma, PTSD, or even attributional style may have had an additional impact.

Secondly, the study relied exclusively on self-report measures. Reassuringly, most variables could be assessed with well-validated instruments. However, the reliability and validity of our single-item measure assessing the number of depressive episodes remains unclear. Although our study shares this limitation with most earlier studies in the field (Nelson et al., in press), future research should include improved measures (e.g., life chart and structured clinical interview).

Thirdly, it remains to be shown whether results from our web-based survey can be replicated using other types of assessment. Reassuringly, earlier research has shown that results of web-based surveys are equivalent to traditional assessment formats (Skitka & Sargis, 2006). However, it cannot be ruled out that our method of recruitment and data collection may have led to a composition of the sample that is different to samples recruited, for example, *via* clinical services. In addition, the relatively large dropout during data collection, albeit typical for web-based surveys, may additionally have contributed to biased sampling.

## Conclusions

Despite these limitations, this study provides novel evidence for the role of emotion regulation difficulties, avoidant attachment, depressogenic attributional style, and PTSD symptom severity as mediators for the link between CM and depression. Future research should combine the psychobiological and the psychological perspectives to examine these vulnerabilities across different levels of analysis.
